# Burnout and job satisfaction among critical care nurses in Saudi Arabia and their contributing factors: A scoping review

**DOI:** 10.1002/nop2.843

**Published:** 2021-03-24

**Authors:** Nawal Alzailai, Louise Barriball, Andreas Xyrichis

**Affiliations:** ^1^ Florence Nightingale Faculty of Nursing, Midwifery and Palliative Care King's College London London UK; ^2^ College of Health Sciences in AL‐Qunfudah Umm AL‐Qura University Mecca Kingdom of Saudi Arabia

**Keywords:** burnout, critical care, ICU, job satisfaction, nurses, Saudi Arabia

## Abstract

**Aim:**

Nurses working in highly demanding areas, such as intensive care units, are more likely to experience burnout and low job satisfaction than nurses working in less demanding areas. This review aims to assess the degree of well‐being of nurses working in critical care settings in Saudi Arabia by evaluating their levels of burnout and job satisfaction, as well as the factors that contribute to them.

**Design:**

A scoping review.

**Method:**

Seven databases were searched for published research that examined the levels and factors of burnout and job satisfaction in intensive care units nurses in the Kingdom of Saudi Arabia, as well as literature in which terminology other than “burnout” or terms related to burnout (such as “stress,” “compassion fatigue” and “moral distress”) were used that were conducted within Saudi Arabia. Data extraction and synthesis were performed by one reviewer and verified by a second reviewer. The present review was undertaken between January 2020 and December 2020.

**Results:**

Eleven studies related to burnout and job satisfaction, and their contributing factors were identified. Evidence from this review indicated that intensive care units nurses in Saudi Arabia are suffering from moderate to high levels of burnout while experiencing only moderate levels of job satisfaction. Three categories of factors were found to be associated with burnout and nursing job satisfaction: intrapersonal, interpersonal and extra‐personal factors. The review highlights important findings for policy and nursing practice that can inform future studies and the development of burnout management strategies.

## 1 INTRODUCTION

Nurses play a major role in providing care in Intensive Care units (ICUs) as members of interprofessional teams, where the care they provide is more complex than that provided elsewhere in the hospital. ICU nurses must respond regularly to high demands to fulfil the many roles that are assigned to them. They work in the ICUs as caregivers, health educators, researchers and unit managers (Aitken et al., 2019). Working in an area that requires such complex multitasking with a heavy workload and the need to provide specialized care for critically ill and highly reliant patients can be overwhelming for nurses. Consequently, occupational burnout may result from the high level of stress involved, which can lead to low job satisfaction and high turnover (Alharbi & Alshehry, 2019). This scoping review aims to determine the level of burnout and job satisfaction and their associated factors in ICU nurses based on the Saudi context.

The phenomenon of burnout has been identified by the World Health Organization as a syndrome resulting from prolonged exposure to workplace stress that has been unsuccessfully managed (World Health Organization [WHO], 2019). Burnout is characterized by three dimensions: (a) emotional exhaustion (EE)—the feeling of depletion of emotional resources and a consumption of energy; (b) depersonalization (DP)—distance from work‐related activities, increased emotional distance, negativism and cynicism towards one's work and feelings of frustration; and (c) reduced personal accomplishment (PA)—reduced work performance associated with a negative work attitude and a feeling of incompetence and ineffectiveness (Maslach et al., 1986; WHO, 2019). Clinical symptoms of burnout include exhaustion, anxiety, irritability, insomnia and emotional instability (Maslach & Leiter, 2006). Healthcare professionals are particularly liable to develop burnout, and the ICU environment is likely to be associated with the experience (Alharbi et al., 2016).

Job satisfaction in nursing profession was defined as the fulfilment of desired needs within the work environment, happiness or gratifying emotional response towards working condition, and job values or equity (Liu et al., 2016). This definition was generated by Liu et al., (2016) using Walker and Avent's approach of concept analysis and based on analysing of multiple theories including, Maslow's hierarchy of needs theory, Herzberg's two‐factor theory and cognitive process of motivation theory. In the literature, burnout and low levels of job satisfaction are usually linked, and suggestive evidence has found that one leads to the other (Alharbi et al., 2016; Khamisa et al., 2015). It is important to investigate related variables, as nurses’ burnout has multiple aspects including, chronic fatigue, moral distress, inefficiency, emotional instability and unexplained nurse turnover (Chang et al., 2018; Whittaker et al., 2018).

## 2 BACKGROUND

In the Kingdom of Saudi Arabia (KSA), nurses of Saudi origin are in short supply and the government recruits many expatriate nurses (constitutes 50% of the nursing workforce in the KSA; Alboliteeh et al., 2017) to cover this shortage (Ministry of Health, 2018). In addition, the nursing profession in Saudi Arabia, which has faced a nursing shortage and challenges in the nursing work environment, has evolved since the development of the Vision 2030 programme that contributes to significant improvements in the areas of health delivery system, nursing, education, trade, communication, technology and science (Alsufyani et al., 2020). However, the demand for nurses in Saudi Arabia is expected to be more than double in the next 10 years, according to predictions (Alsufyani et al., 2020). This means that 150,000 nursing positions will need to be filled by 2030 (Alsufyani et al., 2020). To meet this goal, the nursing shortage and the turnover rate in the profession must be tackled; as such, factors that may affect the quality of the nursing work environment and job satisfaction levels, such as nursing burnout, must be identified and managed. Evidence has shown that nursing burnout levels range from moderate to high among nurses in Saudi Arabia. For instance, Habadi et al., (2018) administered a survey to assess the prevalence of burnout among nurses working in Jeddah, KSA; they reported that, based on their findings, 59.89% of Saudi Arabia's nurses have high EE (37.02 ± 7), 31% have moderate DP (9.32 ± 1.64) and 27% have low PA (25.88 ± 4.5). Moreover, although the country mainly depends on expatriate nurses, little is known about their work experiences, especially those working in emotionally and physically demanding areas like ICUs. Therefore, investigation regarding the job satisfaction and well‐being of ICU nurses in the KSA is important.

Registered nurses in the KSA are qualified with bachelor's degrees, which is achieved by completing a five‐year nursing science programme (Aldossary et al., 2008). Pre‐bachelor's degree level education programmes, such as associate's degree nursing assistant programmes and diploma courses that include two years of theory‐based education and six months of clinical training, were introduced previously in the KSA. These have now been phased out by the Ministry of Higher Education (Almalki et al., 2011) in order to increase enrolment in bachelor's degree nursing programmes. However, pre‐bachelor's degree nurses are still needed, at least for the next few years, if the KSA wishes to address the shortage in the nursing profession. Each level of the nursing workforce has a specific scope of practice within the healthcare team: for example, nursing assistants perform basic, simple nursing procedures that can be time‐ and effort‐consuming; if registered nurses are required to perform these procedures, their ability to perform higher level duties, especially in multitask areas such as ICUs, is hampered (Almalki et al., 2011), which can create stress. In addition, Saudi registered nurses are introduced to the ICU without formal training in critical care skills; they only receive such training as part of their undergraduate study during their internship year (Arabi & Al Shimemeri, 2006). Thus, work‐related stress may be more likely to occur among ICU nurses in Saudia Arabia, particularly among newly graduated nurses who are introduced to this setting without adequate knowledge and skills.

Burnout and job satisfaction among ICU nurses are important variables to investigate. Until recently, two previous reviews were conducted internationally to determine the level of burnout (Friganovic et al., 2019) or job satisfaction (Dilig‐Ruiz et al., 2018) among ICU nurses; however, none of these reviews were designed to include both variables or were aimed to identify the factors associated with these two variables. In Saudi Arabia, multiple studies have provided evidence on burnout, job‐related stressors and job satisfaction among ICU nurses (Batran, 2019), anxiety and stress coping strategies (Alharbi & Alshehry, 2019) and factors that influence ICU nurses' job satisfaction, such as leadership style (Alshahrani & Baig, 2016). However, these findings have not yet been synthesized. Thus, this scoping review examined all available evidence reporting on levels of burnout and job satisfaction among ICU nurses in the Saudi context to inform an understanding of the factors associated with these two variables. In addition, investigation of burnout, job satisfaction and their contributing factors would give appropriate information about the problems and enable the development of appropriate improvement programmes. The present review was designed to answer the following research question: what are the levels of and factors associated with burnout and job satisfaction among ICU nurses in Saudi Arabia?

## 3 METHODS

This systematic scoping review mapped all available evidence on burnout and job satisfaction levels in ICU nurses in Saudi Arabia. The scoping review methodology provides a summary of evidence that exists on a topic in order to reveal the depth and breadth of that topic (Arksey and O'Malley, 2005). These findings are reported based on the Preferred Reporting Items for Systematic Review and Meta‐Analysis Extension for Scoping Review (PRISMA‐ScR), which also guided the protocol for this scoping review (Tricco et al., 2018). The protocol has been developed, and it can be provided upon request from the corresponding author. This scoping review was undertaken between January 2020 and December 2020, and databases were searched for relevant studies from the inception to June 2020.

### 3.1 Eligibility criteria

To select studies, the following inclusion criteria were applied: (a) literature reporting on studies that examined the levels of burnout and job satisfaction of ICU, including both studies that assessed nurses' levels of burnout and/or job satisfaction and studies that identified factors associated with either or both of these two variables; (b) literature documenting studies conducted on nurses working in ICUs (adult ICU, neonatal ICU and paediatric ICU); (c) studies conducted within the context of Saudi Arabia; and (d) studies that used terminology other than burnout or related to burnout, such as exhaustion, stress, compassion fatigue or intention to leave (ITL).

The following exclusion criteria were applied: (a) papers written in languages other than English, due to the time and resources involved in the translation process; and (b) literature that reported on studies that included mixed samples (i.e. departments other than ICUs), where data on burnout or satisfaction of ICU nurses were not presented separately.

### 3.2 Information source and search strategy

A systematic search was performed in the following databases: Medline (Ovid), Embase (Ovid), CINAHL, PsycINFO, Web of Sciences, ScienceDirect Journals (Elsevier) and the Saudi Digital Library. The search was limited to include only articles written in English but without consideration of the study design or date of publication to identify and map all available evident on the topic of interest to determine gaps in knowledge. The systematic search strategy is available in Appendix S1I. To identify additional relevant papers, searches of Google Scholar and the key journals (e.g. *Saudi Critical Care Journal*, *Intensive and Critical Care Nursing Journal*) were conducted. To ensure search saturation, the reference lists of identified studies were scanned to identify any additional relevant studies not captured in the original searches.

### 3.3 Data charting process

Based on the research question and the design of studies identified, a data extraction template was developed. The template was piloted on a sample of included studies to ensure that all relevant data were captured and that resources were not wasted on mining unnecessary data. For each study included, the following data were extracted: study characteristics, such as author, study design, location, year, methods and aim/objective of the study; sample demographic characteristics, such as sample size, gender, age, marital status and years of experience; and main results of the study. Data extraction of all included studies was performed by the lead author (NA), and a sample of these data was checked by the senior author (AX).

### 3.4 Synthesis of results

The extracted data were charted in a spreadsheet to capture all variables related to the scoping review question. The findings from each study were then synthesized, and the key points were highlighted to generate an overall perspective on the data that emerged from the literature. The data were described in relation to the research question and in the context of the overall study objectives. The data are presented using tables and text to summarize and explain the characteristics and findings of each study. One reviewer conducted the initial data synthesis (NA).

## 4 RESULTS

### 4.1 Study selection

**FIGURE 1:**
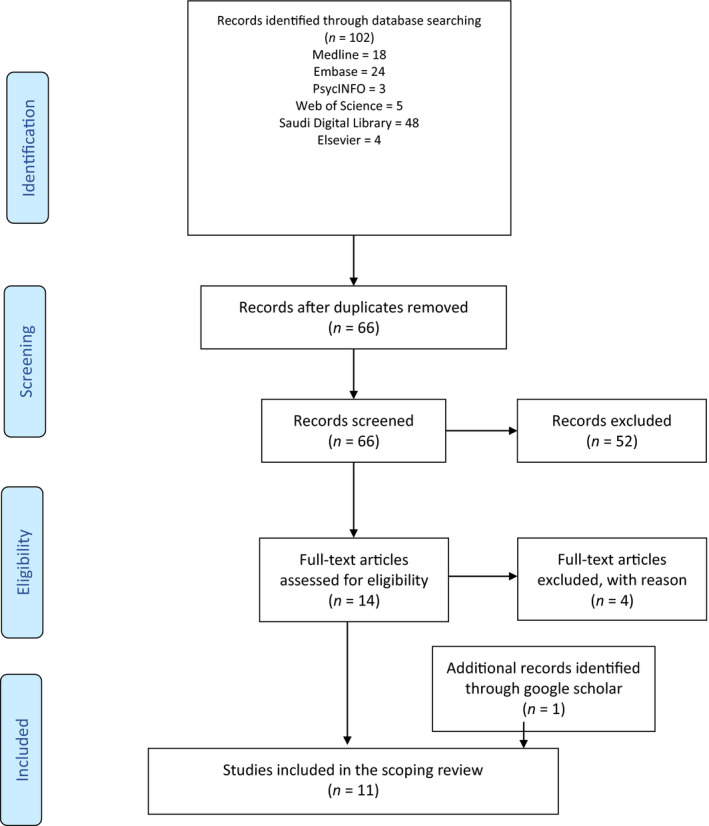
Prisma Flow Diagram (Moher et al., 2009)

A total of 102 studies were identified from the database search; after duplicates were removed, 66 titles and abstracts were screened for eligibility against inclusion and exclusion criteria, which resulted in the exclusion of 52 studies. The full text of the remaining 14 studies was retrieved and screened for eligibility, and 4 studies were found ineligible for the following reasons: participants were recruited from specialties other than nursing (*N* = 1) and from departments other than ICUs (*N* = 3), where results of critical care nurses were not separated. One additional study was identified from Google Scholar. In total, 11 studies met the inclusion criteria and were included in the scoping review. Figure 1 presents a Prisma Flow Diagram illustrating the search results (Moher et al., 2009).

### 4.2 Participant and study characteristics

The sample size of included studies ranged from 135–321, with a total of 1,932 nurses who were working in critical care departments. Studies covered a wide geographical area of Saudi Arabia. Of the 11 paper identified for the review, 4 were based on studies conducted in Riyadh city (the capital of Saudi Arabia), 1 reported on a study conducted in 2 different regions, Najran and Riyadh, and 4 articles were each based on 1 study held in 1 of the following regions: Jeddah (*N* = 1), Tabuk (*N* = 1), Aseer (*n* = 1) and Hail (*N* = 1). The two remaining articles (Alharbi et al., 2019 and Alharbi et al., 2020) reported on the same study, which was conducted in 2 different regions, Qasim and Hail. Duplicate publications may be difficult to manage, and their inclusion may introduce bias (von Elm et al., 2004); however, both articles were included, as each focused on a different aim and presented different outcomes. Moreover, similarities across the two papers were acknowledged, and the sample size of one paper was not added to the total sample count for the review. This was done to avoid double counting and to allow data from the two papers to be pieced together (Shamseer et al., 2015). All included studies adopted a cross‐sectional design (*N* = 11). Study characteristics are presented in Table 1, and results relating to scores of burnout dimension with reported associations are presented in Table 2

**TABLE 1:** Characteristics of included studies (ordered alphabetically by author name)

Author/year/ region in Saudi Arabia	Study design/methods	Population and setting	Aim	Main results
Abumayyaleh et al., (2016) Riyadh	Cross‐sectional design	Nurses working in adult ICU Setting: Teaching university hospital in Riyadh Sample size: 135 nurses (98.5% female, 1.5% male) Nationality: not determined (ND) Median age: 33.5 (age range 24‒54) Years of ICU experience: ND (*M = *11.3, *SD* = 4.9 years) Working hours: ND Marital status: 85.2% married Level of education: 87.4% diploma level Medical illness history: ND	(a) To determine the level of moral distress among critical care nurses and (b) to identify relationships between moral distress and turnover intention	A moderate level of moral distress was reported among ICU nurses
Alasmari and Douglas (2012) Jeddah	Cross‐sectional design	Nurses working across six critical care units (medical, surgical, NICU, PICU, coronary care unit and high dependency unit) Setting: King Abdul‐Aziz university hospital (KAUH) Sample size: 182 nurses (94% female, 6% male) Nationality: ND Median age: 33.3 (age range 23‒56) Years of ICU experience: ND (*M* = 5.1 years, *SD* = 4.2) Working hours: ND Marital status: 76% married Level of education: 78.4 diploma level Medical illness history: ND	(a) To determine the level of satisfaction of ICU nurses in Saudi Arabia and (b) to examine the relationship between satisfaction of ICU nurses and their intention to leave (ITL)	The results showed high levels of job satisfaction among nurses (*M* = 3.75, *SD* = 0.34), and overall ITL was moderate (*M* = 10.69, *SD* = 3.34).
Alharbi and Alshehry (2019) Riyadh	Cross‐sectional design	Nurses working in cardiac (67%) and surgical adult ICU and paediatric ICU (PICU) Setting: Two tertiary training hospitals Sample size: 154 nurses (96.1% female, 3.9% male) Nationality: ND Median age: 35.5 (age range 20‒55) Years of ICU experience: 33% range 6‒10 years Working hours: ND Marital status: ND Level of education: 51% bachelor's degree Medical illness history: ND	To examine level of perceived stress and coping strategies among critical care nurses in Saudi Arabia	Most participants reported a moderate level of stress (87%). Nurses working in cardiac ICU indicated significantly higher levels of stress (*p* = .025) compared to nurses from surgical ICU, [*M* (*SD*) = 18.18 (3.88) vs. 6.17 (3.21)].
Alharbi et al., (2016) Hail	Cross‐sectional design	Nurses working in critical care units (ICU, PICU, CCU, NICU and emergency department) Setting: three government hospitals in Hail Sample size: 150 nurses (87% female, 13% male) Nationality: 62% Saudi, 38% non‐Saudi Median age: ND (age range 20‒45) Years of ICU experience: 75% less than 5 years Working hours: ND Marital status: 52% single Level of education: 66% diploma level Medical illness history: ND	To examine the prevalence of burnout and job satisfaction and their contributing factors among Saudi critical care nurses	Nurses in this study reported high levels of burnout. The majority of nurses experienced high levels of burnout in the area of emotional exhaustion (84%, *N* = 126, *M* = 35.19, *SD* = 8.92) and depersonalization (77% *N* = 115, *M* = 16.34, *SD* = 5.24), while only 42% reported high levels in personal accomplishments.
Alharbi et al., (2019) Qassim and Hail	Cross‐sectional design	Nurses working in adult ICU (59.5%) and NICU Setting: Four government hospitals from different regions Sample size: 321 nurses (88% female, 12% male) Nationality: 63% non‐Saudi Median age: ND (age range 20‒56) Years of ICU experience: 39% range 2‒5 years Working hours: 8 hr shifts (92.8%) Marital status: ND Level of education: 63% bachelor's degree Medical illness history: ND	To determine the prevalence of compassion fatigue (CF) and association between CF and demographic characteristics as indicated by level of burnout, secondary stress syndrome (STS) and compassion satisfaction (CS) in critical care nurses in Saudi Arabia	Average scores for CS were reported for 77% of nurses; very low scores for CS were reported for 20%, which would indicate the presence of fertile ground for the development of CF. A significant correlation was indicated between CS and nationality (*p* = .011) (Saudi nurses having higher subscale scores than non‐Saudi) and years of ICU experience (*p* = .03) (experience less than 1 year and more than 10 years scored highest among the groups in terms of CS).
Alharbi et al., (2020) Qassim and Hail	cross‐sectional design	Nurses working in adult ICU (59.5%) and NICU Setting: Four government hospitals from different regions Sample size: 321 nurses (88% female, 11.8% male) Nationality: 63% non‐Saudi Median age: ND (age range 20‒56) Years of ICU experience: 39% range 2‒5 years Working hours: 8 hr shifts (92.8%) Marital status: ND Level of education: 63% bachelor's degree Medical illness history: ND	^(1)^To identify the correlation between three nurse‐sensitive indicators (pressure injuries, patient falls and medication errors) and the level of CF	The majority of participants indicated average CF on the professional Quality of Life scale (proQol).
Alshahrani and Baig (2016) Aseer	Cross‐sectional design	Nurses working in critical care units Setting: Aseer Central Hospital (ACH) Sample size: 160 nurses (98% female, 2% male) Nationality: ND Median age: ND (age range 22‒58) Years of ICU experience: ranged from 6 months–17 years Working hours: 8 hr shifts Marital status: 57% married Level of education: 79% bachelor's degree Medical illness history: ND	(a) To determine the level of job satisfaction in critical care nurses at a tertiary hospital in Saudi Arabia and (b) to evaluate the effect of transformational (TF) and transactional (TA) leadership style of head nurses on job satisfaction of staff nurses	Nurses were moderately satisfied (*M* = 3.40, *SD* = 0.57). Nurses working under TF leadership style demonstrated significantly higher levels of job satisfaction (*p* ˂ .05).
Awajeh et al., (2018) Riyadh	Cross‐sectional design	Nurses working in adult ICU Setting: King Saud Medical City (KSMC) Sample size: 270 nurses (97% female, 3% male) Nationality: ND Median age: 30.9 (age range 25‒34 for 89%) Years of ICU experience: 48% more than 5 years Working hours: 12 hr shifts Marital status: 60% married Level of education: 81.5% bachelor's degree Medical illness history: 85.9% no medical history	To examine the degree of burnout among ICU staff nurses and different determinants	Severe burnout among most ICU nurses (65%, *N* = 178 nurses) in all three dimensions—depersonalization, emotional exhaustion and personal achievement—with highest level in the area of emotional exhaustion *p* (.003).
Batran (2019) Najran and Riyadh	Cross‐sectional design	Nurses working in adult ICU or neonatal ICU Setting: Four government hospitals (three in Najran and one in Riyadh region) Sample size: 213 nurses (90.1% female, 9.9% male) Nationality: ND Median age: 55.9% were 30 or under Year of ICU experience: 52% more than 5 years Working hours: ND Marital status: 50.3% single Level of education: 62% bachelor's degree Medical illness history: ND	(a) To identify factors contributing to stress among critical care nurses in Saudi Arabia and (b) to determine the effect of stressors on the mental and physical health of nurses	The highest average scores were reported for workload from among the work‐related stressors (*M* = 59.4, *SD* = 14.67); the second highest source of stress was lack of support (*M* = 55.32, *SD* = 19.13) and the third was dealing with death and dying (*M* = 54.79, *SD* = 17.42).
Mari et al., (2018) Tabuk	Cross‐sectional design	Nurses working in different ICU Settings (adult ICU, PICU, NICU, coronary care unit) Setting: Main government hospital in the region Sample size: 190 nurses (87% female, 13% male) Nationality: ND Median age: ND (age range 20‒30 for 65%) Years of ICU experience: 58% less than 5 years Working hours: ND Marital status: ND Level of education: 80% bachelor's degree Medical illness history: ND	To examine the prevalence of job satisfaction and factors that affect nurses’ job satisfaction	The level of job satisfaction was intermediate among ICU nurses.
Muhawish et al., (2019) Riyadh	Cross‐sectional design	Nurses working in adult ICU Setting: Tertiary teaching hospital Sample size: 150 nurses (93.3% female, 6.7% male Nationality: 4.6% Saudi, 95.4% non‐Saudi Median age: ND Years of ICU experience: ND Working hours: 12 hr shifts Marital status: ND Level of education: 70% diploma level Medical illness history: ND	To determine job‐related stressors and their effects on job satisfaction of ICU nurses working in Saudi Arabia	The most stressful factors among ICU nurses were conflicts on the job, criticism and discrimination; this was in the area that included dealing with abuse from patients' families (*M* = 3.86, *SD* = 0.34).

**TABLE 2:** Actual scores recorded for the different burnout dimensions with reported association (contributing factors) and correlation value

STUDY	Burnout dimension	Scores (mean, *SD*)	Reported association	Tool used	Correlation, *p* value
Abumayyaleh et al., (2016)	Moral distress	Mean = 1.32, *SD* = 0.80	Age. Years of ICU experience.	1‐ Hamric MDS	*r* = .17, *p* ˂ .05 *r* = .18, *p* ˂ .05
Alasmari and Douglas (2012)	Job satisfaction & ITL	JS (mean = 3.75, *SD* = 0.34), ITL (mean = 10.69, *SD* = 3.34)	Professional support. Parental status. Workload. Pay.	MJS ITL scale drawn from Price and Mueller (1981)	*r* = −.28, *p* = .001 *t* = 2.13, *p* = .03 *r* = −.28, *p* = .008 *r* = −.22, *p* = .004
Alharbi and Alshehry (2019)	Stress	Mean = 17.71, *SD* = 3.76	Work department (cardiac vs. surgical ICU)	PSS	*F* = 3.48, *p* =.033
Alharbi et al., (2016)	Burnout (EE, DP, PA) Job satisfaction	EE (mean = 35.19, *SD* = 8.92), DP (mean = 16.34, *SD* = 5.24), PA (mean = 33.91, *SD* = 8.03)JS (mean = 108.9, *SD* = 11.4)	Contingent rewards communication	MBI JSS	*r* = −.34, *p* ˂ .001 *r* = −.29, *p* ˂ .001
Alharbi et al., (2019); Alharbi et al., (2020)	Compassion satisfaction & burnout	Mean = 35.39, *SD* = 7.26 Mean = 31.74, *SD* = 5.35	Nationality. Years of ICU experience	ProQoL	*t* = 4.348, *p* ˂ .001 *F* = 2.407, *p* = .03
Alshahrani and Baig (2016)	Job satisfaction	Mean = 3.40, *SD* = 0.57	Leadership style (professional support)	JSS	*r* = .46. *p* =.007
Awajeh et al., (2018)	Burnout	Nd	Age. Marital status. Presence of medical illness	MBI	Nd, *p* ˂ .05 Nd, *p* = .015 Nd, *p* = .021
Batran (2019)	Sources of work‐related Stress	Workload (mean = 59.43, *SD* = 14.67) Lack of support (mean = 55.32, *SD* = 19.13) Dealing with death and dying (mean = 54.79, *SD* = 17.42)	Gender (male)	Self‐developed questioner to assess work‐related stressors	*r* = ND, *p* = .001
Mari et al., (2018)	Job satisfaction	Mean = 3.84, *SD* = 0.49	Communication Supervision	JSS	*r* = .418, *p* ˂ .001 *r* = .250, *p* ˂ .005
Muhawish et al., (2019)	Job satisfaction	Mean = 3.8, *SD* = 0.43	‐	JSS ENSS	‐

Abbreviations: DP, depersonalization; EE, emotional exhaustion; ITL, intention to leave; JSS, job satisfaction survey; MBI, maslach burnout inventory; MDS, Moral Distress Scale; MJS, measure job satisfaction; PA, personal accomplishment; ProQol, Professional Quality of Life; PSS, Perceived Stress Scale; ENSS, Expanded Nursing Stress Scale.

### 4.3 Quality assessment of included studies

A methodological quality assessment was completed for each study in this review. The National Heart, Lung and Blood Institute (NIH) quality assessment tool was used to assess the quality of the quantitative studies. Eleven studies were assessed against the NIH quality assessment tool for observational cohort and cross‐sectional studies (see Appendix S1II). As the tools do not suggest scoring systems, the overall quality rating for the studies focused on key concepts to assess the internal validity with consideration of the methodological flaws that may result in a potential risk of bias. To help determine the overall quality judgement for included studies, a simple scoring system was developed by the first author, which was reviewed by the second author, and support for judgement statements was provided (see Appendix S1III). Within this review, seven studies were rated as fair (moderate risk of bias) and four were assigned poor quality ratings (high risk of bias).

### 4.4 Level of burnout and dissatisfaction among ICU nurses in Saudi Arabia

Five studies reported on different aspects of burnout, across these studies, three reported moderate levels of burnout (Abumayyaleh et al., 2016; Alharbi & Alshehry, 2019; Alharbi et al., 2019) and two indicated severe levels of burnout (Alharbi et al., 2016; Awajeh et al., 2018). Awajeh et al., (2018) found that the majority of nurses working in critical care departments in their sample (65% of 270 nurses) experienced high levels of burnout in all dimensions (depersonalization, emotional exhaustion and personal achievement). Another study reported 85% of 126 Saudi nurses working in ICUs had high levels of emotional exhaustion (Alharbi et al., 2016).

Job satisfaction as investigated in five of the identified studies (*N* = 650) (Alasmari & Douglas, 2012; Alharbi et al., 2016; Alshahrani & Baig, 2016; Mari et al., 2018; Muhawish et al., 2019) ranged from moderate to low (mean = 3.4, *SD* = 0.57–mean = 4.24, *SD* = 1.66). Three studies reported moderate satisfaction among the nurses in their sample populations (Alharbi et al., 2016; Alshahrani & Baig, 2016; Mari et al., 2018), and one reported overall low satisfaction among the nurses studied who worked in adult ICUs, with the lowest satisfaction related to pay (Muhawish et al., 2019). Only one study indicated high levels of job satisfaction with a mean score of 3.75 (on a 5‐point scale where 1 indicated the lowest satisfaction level and 5 indicated the highest level); however, the results also showed a moderate rate of ITL among the ICU nurses in the study (Alasmari and Douglas, 2012).

### 4.5 Factors contributing to burnout and dissatisfaction in ICU nurses in Saudi Arabia

**FIGURE 2:**
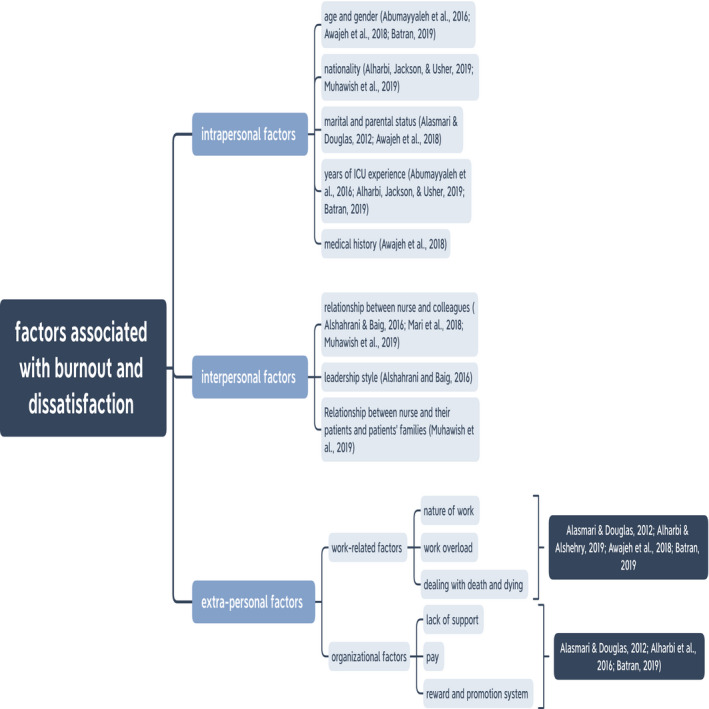
Three Factors Associated with ICU Nurses’ Burnout and Low Job Satisfaction in Saudi Arabia

Data from included studies were charted in a spreadsheet and then narratively synthesized. This data synthesis generated three main factors associated with the prevalence of burnout and lack of job satisfaction in ICU nurses in Saudi Arabia: intrapersonal factors, interpersonal factors and extra‐personal factors (see Figure 2).

## 5 DISCUSSION

In this scoping review, we provided a comprehensive overview of the available evidence that assess the level of burnout and satisfaction in ICU nurses in the Saudi Arabia and their contributing factors. It also provided an insight to the methodological quality of individual studies included in the analysis. Based on the finding and the methodologies of included studies, we propose recommendations for practice and future research.

### 5.1 Main findings and recommendations

**FIGURE 3:**
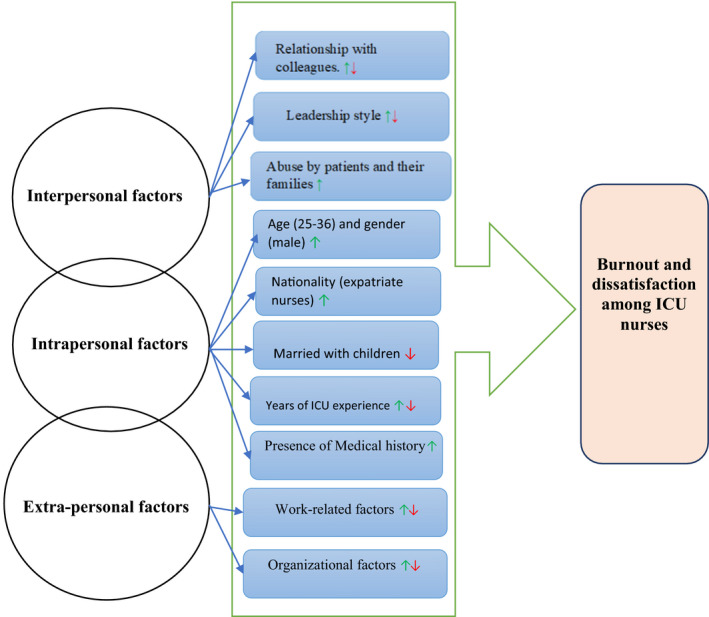
Factors associated to burnout and dissatisfaction in Saudi ICU nurses with their positive and /or negative effect

All of the studies included in this review focused on the level of burnout, job satisfaction and the factors contributing to both. The evidence showed moderate to high levels of burnout and moderate levels of job dissatisfaction among ICU nurses in Saudi Arabia. The findings also highlighted the association between burnout, job satisfaction and three contributing factors: (a) intrapersonal factors, (b) interpersonal factors and (c) extra‐personal factors. These factors work as multidimensional variables that can increase or decrease levels of burnout and dissatisfaction in ICU nurses (see Figure 3). As presented in Figure 3, the intrapersonal factors were placed in the middle of the three factors to show that intrapersonal factors could have a mediating effect on the interpersonal and extra‐personal factors. The green arrows (↑) in Figure 3 indicate positive (increase) effects of the variable on burnout and dissatisfaction while red arrows (↓) represent a negative (decrease) effect; the ↓↑ symbol indicates that the variable can have either effect (positive and/or negative).

This review found that younger nurses from 25–34 years of age experienced severe burnout compared to senior nurses (Awajeh et al., 2018). This finding is consistent with the meta‐analysis conducted by Gómez‐Urquiza et al., (2017), which assembled data from 51 studies (48% were done in Europe, 31% in America and 21 in Asia). The consensus of those studies was that younger nurses were at a high risk of developing burnout, and there was a statistically significant correlation between younger age and two dimensions of burnout: [EE (*p* ˂ .05) and DP (*p* ˂ .001)].

This finding could be because the ICU is physically and mentally exhausting area, particularly for younger nurses who have less knowledge and experience in critical care than older and more experienced staff. This age‐related difference applies to recently graduated nurses introduced to the ICU setting without sufficient training in critical care. This finding is crucial for identifying those at high risk for burnout. In addition, it highlights the importance of support for younger nurses to reduce their occupational stress and prevent burnout.

In contrast, some studies found that senior nurses with extensive ICU experience were exposed to prolonged work‐related stress and are, therefore, more liable to burnout and wanting to leave their job (Abumayyaleh et al., 2016; Batran, 2019). A possible explanation for these contradictory findings is that they were generated in different studies with different participants. Each participant has their unique intrapersonal characteristics that can affect an individual's response to stress and their coping strategies. Longitudinal studies that investigate the link between a low sense of well‐being and personal characteristics may be able to determine how the contributing factors affect different members of the nursing team. Longitudinal studies may also help identify the professional consequences of burnout, including patient outcomes, quality of care, safety and cost.

This review found that both discrimination and abuse may be associated with nurses’ burnout (Muhawish et al., 2019). Therefore, an appropriate mentoring and preventive measure should be adopted to manage discrimination and abuse among nurses with a clear and easily accessible institutional procedure to report any mistreatment. Countries like Saudi Arabia, which recruit nurses from different nationalities and backgrounds, must make an extra effort to ensure that any discrimination or abuse in their health organizations is reduced. In a survey of 198 multinational nurses working in Saudi Arabia, Al‐Turki et al., (2010) reported that the Non‐Saudi nurses were significantly more liable to EE (27.3 ± 12.1vs. 21.6 ± 2.9) than Saudi nurses (*p* = .004; 95% CI: <9.64). Moreover, the literature search did not identify records regarding discrimination among nurses in both Saudi and international context. Further research must be conducted to determine the prevalence, sources and consequences of discrimination and abuse in the nursing profession in Saudi Arabia.

Other findings of this review were the effects of work overload (*p* = .01) and death and dying (*p* ˂ .05) on burnout and job satisfaction (Alasmari & Douglas, 2012; Batran, 2019). In a cross‐sectional study, Cishahayo et al., (2017) reported a statistically significant association between burnout and work overload among ICU nurses (*p* ˂ .05). Further research is needed to determine the prevalence and source of excessive workload for ICU nurses. Some quantitative studies suggest that long working hours and inadequate staffing contribute to high workload (Cishahayo et al., 2017). However, future qualitative research that promotes a greater understanding of the nurses’ perception of high workload and its adverse effects should be considered.

Confronting death and dying was considered by previous studies to be one of the significant risk factors contributing to ICU nurse burnout. Thus, resources and educational effort are required to improve coping strategies and the grief response of ICU nurses. This study provides evidence of the association between burnout and a nurse's experience with a patient's death. However, the influence of grief on the quality of nursing care in the ICU requires further research.

The current review also identified several organizational risk factors influencing burnout and the satisfaction of ICU nurses about support, pay, rewards and promotions (Alasmari & Douglas, 2012; Alharbi et al., 2016; Batran, 2019). However, a study by McHugh and Ma (2014) found no statistically significant association between burnout, job dissatisfaction and pay. This disconnect indicated that nurses might require more than higher pay to improve their wellness and satisfaction. Instead, a comprehensive organizational support system for ICU nurses that consider all of the variables affecting nurse burnout should be established.

An intervention to prevent/reduce and manage burnout of ICU nurses need to be established to address modifiable factors leading to burnout and to provide nurses with a better understanding of how to cope with stress in the critical care environment.

### 5.2 Strengths and limitations of this review

This study makes a significant contribution to knowledge on ICU nurse burnout and job satisfaction by being the first review of research, practices and other factors contributing to burnout and job dissatisfaction among ICU nurses in Saudi Arabia. The study approach included a comprehensive search strategy using the PRISMA‐ScR guidance. A quality assessment was conducted for all included studies to determine the strength of the evidence. This scoping review provided a comprehensive understanding of the factors associated with burnout and job dissatisfaction in Saudi ICU nurses. Although data were extracted only from studies in the Saudi context, findings can have resonance for similar contexts such as other Gulf countries. This review identified gaps in knowledge and opportunities for future studies.

Despite these strengths, this study should also be considered in light of its limitations. All of the included studies in this review were at moderate or high risk of bias mainly because of insufficient statistical power and length of follow‐up to generate strong evidence concerning the factors contributing to nurses’ burnout and dissatisfaction in ICU. The review may be subject to language bias because it only included studies that were written in English. Scoping reviews have inherent limitations because they seek to provide breadth rather than depth of information. However, this method was considered appropriate, given that our objective was to identify the evidence for burnout and job dissatisfaction among Saudi ICUs. Seven databases were reviewed, but not grey literature; therefore, some relevant unpublished studies may have been overlooked. There was a notable absence of qualitative studies that could provide insight into the nurses’ perceptions and opinions about the factors contributing to their burnout.

## 6 CONCLUSION

This review identified 11 studies about burnout and job dissatisfaction among Saudi ICU nurses. The studies identified a high level of burnout among Saudi ICU nurses that ranged from moderate to high, with only an average level of job satisfaction. The primary factors associated with burnout and dissatisfaction fell into three categories: intrapersonal, interpersonal and extra‐personal. A synthesis of the findings from the reviewed studies identified several recommendations for research and the involvement of stakeholders to promote the understanding of the factors and consequences of burnout in the ICU.

## CONFLICT OF INTERESTS

No conflict of interest.

## ETHICAL APPROVAL

No ethical approval was required for this study.

## Supporting information

Appendix S1

## Data Availability

The data that support the findings of this study are available from the corresponding author upon reasonable request.
