# Supplementary material for: Burnout and job satisfaction among critical care nurses in Saudi Arabia and their contributing factors: A scoping review
Source: Nurs Open. 2021 Mar 24;8(5):2331–44. doi: 10.1002/nop2.843 (PMC8363385; doi:10.1002/nop2.843)
Supplement: Supplementary file 1 — Appendix S1 [file NOP2-8-2331-s001.docx]

**Appendices**

**Appendix I**

Database: Embase Classic+Embase <1947 to 2020 June 01>

Search Strategy:

--------------------------------------------------------------------------------

1 nursing staff.mp. or exp nursing staff/ (79421)

2 nurs*.mp. (826226)

3 nursing.mp. (623401)

4 nurses.mp. (223128)

5 1 or 2 or 3 or 4 (826226)

6 critical care.mp. or exp intensive care/ (736395)

7 ICU.mp. (116053)

8 ITU.mp. (2501)

9 PICU.mp. (10403)

10 neonatal intensive care.mp. (29932)

11 NICU.mp. (17901)

12 Pediatric intensive care.mp. (13921)

13 6 or 7 or 8 or 9 or 10 or 11 or 12 (821875)

14 burnout.mp. or exp burnout/ (20983)

15 turnover.mp. (127497)

16 intention to leave.mp. (534)

17 moral distress.mp. (1156)

18 stress*.mp. (1402121)

19 compassion fatigue.mp. or exp compassion fatigue/ (1273)

20 emotional exhaustion.mp. or exp emotional stress/ (23451)

21 exhaust*.mp. (92735)

22 emotional labo?r.mp. (471)

23 job satisfaction.mp. or exp job satisfaction/ (32012)

24 14 or 15 or 16 or 17 or 18 or 19 or 20 or 21 or 22 or 23 (1634631)

25 Saudi arabia.mp. or exp Saudi Arabia/ (25900)

26 exp Saudi/ (1552)

27 KSA.mp. (1684)

28 25 or 26 or 27 (27469)

29 5 and 13 and 24 and 28 (21)

***************************

**Appendix II: Critical appraisal approach of quantitative included studies (by NIH quality assessment tool for observational cohort and cross-sectional studies)**

|  | **Critical appraisal approach of quantitative included studies (by NIH quality assessment tool for observational cohort and cross-sectional studies)** | | | | | | | | | | | | | |  |
| --- | --- | --- | --- | --- | --- | --- | --- | --- | --- | --- | --- | --- | --- | --- | --- |
| **Author/ year** | **Criteria** | | | | | | | | | | | | | | **Overall quality rating** |
|  | 1. Was the research question or objective in this paper clearly stated? | 2. Was the study population clearly specified and defined? | 3. Was the participation rate of eligible persons at least 50%? | 4. Were all the subjects selected or recruited from the same or similar populations (including the same time period)? Were inclusion and exclusion criteria for being in the study prespecified and applied uniformly to all participants? | 5. Was sample size justification, power description, or variance and effect estimate provided? | 6. For the analyses in this paper, were the exposure(s) of interest measured prior to the outcome(s) being measured? | 7. Was the timeframe sufficient so that one could reasonably expect to see an association between exposure and outcome if it existed? | 8. For exposures that can vary in amount or level, did the study examine different levels of the exposure as related to the outcome (e.g. categories of exposure or exposure measured as a continuous variable)? | 9. Were the exposure measures (independent variables) clearly defined, valid, reliable and implemented consistently across all study participants? | 10. Was the exposure(s) assessed more than once over time? | 11. Were the outcome measures (dependent variables) clearly defined, valid, reliable and implemented consistently across all study participants? | 12. Were the outcome assessors blinded to the exposure status of participants? | 13. Was loss to follow-up after baseline 20% or less? | 14. Were key potential confounding variables measured and adjusted statistically for their impact on the relationship between exposure(s) and outcome(s)? |  |
| Alasmari & Douglas (2012) | yes | yes | yes | yes | yes | no | no | yes | yes | no | yes | NA^(1)^ | NA^(2)^ | yes | Fair |
| Alshahrani & Baig (2016) | yes | yes | yes | yes | no | no | no | yes | yes | no | yes | NA (self-reported scale) | NA | yes | Poor |
| Abumayyaleh et al. (2016) | yes | yes | yes | yes | yes | no | no | yes | yes | no | yes | NA | NA | yes | Fair |
| Alharbi et al. (2016) | yes | yes | yes | yes | yes | no | no | yes | yes | no | yes | NA | NA | yes | Fair |
| Mari et al. (2018) | yes | yes | yes | yes | no | no | no | yes | yes | no | yes | NA | NA | yes | Poor |
| Awajeh et al. (2018) | yes | yes | yes | yes | yes | no | no | yes | yes | no | yes | NA | NA | yes | Fair |
| Alharbi, Jackson, & Usher (2019) | yes | yes | yes | yes | yes | no | no | yes | yes | no | yes | NA | NA | yes | Fair |
| Muhawish et al. (2019) | yes | yes | yes | yes | no | no | no | yes | yes | no | yes | NA | NA | yes | Poor |
| Alharbi & Alshehry (2019) | yes | yes | yes | yes | yes | no | no | yes | yes | no | yes | NA | NA | yes | Fair |
| Batran (2019) | yes | yes | yes | yes | no | no | no | yes | yes | no | yes | NA | NA | yes | Poor |
| Alharbi, Jackson, & Usher (2020) | yes | yes | yes | yes | no | no | no | yes | yes | yes | yes | NA | NA | yes | Fair |

NA= Not available

1. This item was marked “NA” for all included studies and was not counted negatively toward the quality of the studies because blinding was not possible since all the studies used self-reported scales to assess the outcome of the participants.
2. Cross-sectional studies are limited in follow-up rate; therefore, this item was marked as “NA” and was not counted negatively toward the quality of the studies.

Based on the NIH quality assessment tool, the overall quality rating for included studies focused on key concepts (14 criteria) to assess the internal validity of the studies rather than having a scoring system intended to create a list of scores to simply summarise (or sum up) to judge the quality (NIH, 2014). In this review the studies that met all fourteen criteria were marked as being of good quality, studies that failed to meet up to three of the criteria were considered as being of fair quality and studies that failed to meet more than three criteria were judged as being of poor quality. In addition, more attention was given to the study design and to the methodological flow that may result in a potential risk of bias, such as selection of study population, justification of sample size and validity and reliability of outcome measures. Support for the judgment statements is given in Appendix III.

**Appendix III: Risk assessment of included studies**

| Study | Overall judgment | Bias domain | Support for judgment |
| --- | --- | --- | --- |
| Alasmari & Douglas (2012) | Fair | - Sufficient timeframe to see effect - Repeated exposure assessment - Blinding of outcome assessor | Exposer and outcome assessed in same timeframe and only once  Blinding of outcome assessor not possible due to use of self-reported scale |
| Alshahrani & Baig (2016) | Poor | - Sample justification - Sufficient timeframe to see effect - Repeated exposure assessment - Blinding of outcome assessor | Researchers presented reasons for recruiting participants; however, they did not identify sample size needed to detect hypothesised difference in outcome (sample size estimation and statistical power)  Blinding of outcome assessor not possible due to use of self-reported scale  Exposure and outcome assessed in same timeframe and multiple assessments for exposer was not applicable |
| Abumayyaleh et al., (2016) | Fair | - Sufficient timeframe to see effect - Repeated exposure assessment - Blinding of outcome assessor | Exposer and outcome assessed in same timeframe and only once  Blinding of outcome assessor not possible due to use of self-reported scale |
| Alharbi et al., (2016) | Fair | - Sufficient timeframe to see effect - Repeated exposure assessment - Blinding of outcome assessor | Due to natural flow in cross-sectional design, exposure and outcome assessed in same timeframe and exposure for each participant assessed once during course of study  Blinding of outcome assessor not possible due to use of self-reported scale |
| Mari et al., (2018) | Poor | - Sample justification - Sufficient timeframe to see effect - Repeated exposure assessment - Blinding of outcome assessor | Reasons for sample selection presented, but sample size estimation and statistical power not reported  Exposure and outcome assessed in same timeframe and exposure assessed only once  Blinding of outcome assessor not possible due to use of self-reported scale |
| Awajeh et al., (2018) | Fair | - Sufficient timeframe to see effect - Repeated exposure assessment - Blinding of outcome assessor | Exposer and outcome assessed in same timeframe and only once  Blinding of outcome assessor not possible due to use of self-reported scale |
| Alharbi, Jackson, & Usher (2019) | Fair | - Sufficient timeframe to see effect - Repeated exposure assessment - Blinding of outcome assessor | Exposer and outcome assessed in same timeframe and only once  Blinding of outcome assessor not possible due to use of self-reported scale |
| Muhawish et al., (2019) | Poor | - Sample justification - Sufficient timeframe to see effect - Repeated exposure assessment - Blinding of outcome assessor | Reasons for sample selection presented, but sample size estimation and statistical power not reported  Exposer and outcome assessed in same timeframe and only once  Blinding of outcome assessor not possible due to use of self-reported scale |
| Alharbi & Alshehry (2019) | Fair | - Sufficient timeframe to see effect - Repeated exposure assessment - Blinding of outcome assessor | Exposer and outcome assessed in same timeframe and only once  Blinding of outcome assessor not possible due to use of self-reported scale |
| Batran (2019) | Poor | - Sample justification - Sufficient timeframe to see effect - Repeated exposure assessment - Blinding of outcome assessor | Reasons for sample selection presented, but sample size estimation and statistical power not reported  Exposer and outcome assessed in same timeframe and only once  Blinding of outcome assessor not possible due to use of self-reported scale |
| Alharbi, Jackson, & Usher (2020) | Fair | - Sample justification - Sufficient timeframe to see effect - Blinding of outcome assessor | Reasons for sample selection presented, but sample size estimation and statistical power not reported  Exposure and outcome assessed in same timeframe; however, assessment of exposure repeated three times for each participant over three-month period. |
